# In vitro evaluation of genipin-crosslinked gelatin hydrogels for vocal fold injection

**DOI:** 10.1038/s41598-023-32080-y

**Published:** 2023-03-29

**Authors:** Wan-Chiew Ng, Yogeswaran Lokanathan, Mh Busra Fauzi, Marina Mat Baki, Ani Amelia Zainuddin, Shou Jin Phang, Mawaddah Azman

**Affiliations:** 1 Department of Otorhinolaryngology-Head and Neck Surgery, Faculty of Medicine, Universiti Kebangsaan Malaysia, 56000 Kuala Lumpur, Malaysia; 2 Centre for Tissue Engineering and Regenerative Medicine, Faculty of Medicine, Universiti Kebangsaan Malaysia, 56000 Kuala Lumpur, Malaysia; 3 Department of Obstetrics and Gynaecology, Faculty of Medicine, Universiti Kebangsaan Malaysia, 56000 Kuala Lumpur, Malaysia; 4 Department of Biomedical Science, Faculty of Medicine, Universiti Malaya, 50603 Kuala Lumpur, Malaysia

**Keywords:** Mesenchymal stem cells, Cell delivery, Protein delivery, Regenerative medicine, Stem-cell biotechnology, Tissue engineering, Bioinspired materials, Biomaterials - cells, Gels and hydrogels, Polymers

## Abstract

Glottic insufficiency is one of the voice disorders affecting all demographics. Due to the incomplete closure of the vocal fold, there is a risk of aspiration and ineffective phonation. Current treatments for glottic insufficiency include nerve repair, reinnervation, implantation and injection laryngoplasty. Injection laryngoplasty is favored among these techniques due to its cost-effectiveness and efficiency. However, research into developing an effective injectable for the treatment of glottic insufficiency is currently lacking. Therefore, this study aims to develop an injectable gelatin (G) hydrogel crosslinked with either 1-ethyl-3-(3-dimethylaminpropyl)carbodiimide hydrochloride) (EDC) or genipin (gn). The gelation time, biodegradability and swelling ratio of hydrogels with varying concentrations of gelatin (6–10% G) and genipin (0.1–0.5% gn) were investigated. Some selected formulations were proceeded with rheology, pore size, chemical analysis and in vitro cellular activity of Wharton's Jelly Mesenchymal Stem Cells (WJMSCs), to determine the safety application of the selected hydrogels, for future cell delivery prospect. 6G 0.4gn and 8G 0.4gn were the only hydrogel groups capable of achieving complete gelation within 20 min, exhibiting an elastic modulus between 2 and 10 kPa and a pore size between 100 and 400 μm. Moreover, these hydrogels were biodegradable and biocompatible with WJMSCs, as > 70% viability were observed after 7 days of in vitro culture. Our results suggested 6G 0.4gn and 8G 0.4gn hydrogels as potential cell encapsulation injectates. In light of these findings, future research should focus on characterizing their encapsulation efficiency and exploring the possibility of using these hydrogels as a drug delivery system for vocal fold treatment.

## Introduction

The reported incidence of voice disorders ranges from 6 to 23% in children and from 12 to 35% in adults^1^. Voice disorders occur when the vocal folds are unable to make firm contact during phonation. Glottic insufficiency has been attributed to multiple causes, including vocal fold paralysis, paresis and atrophy. This condition increases the likelihood of aspiration and phonation difficulties^2,3^. Common treatments for improving the clinical outcome of glottic insufficiency include nerve repair, reinnervation, implant placement and injection laryngoplasty^4^. Injection laryngoplasty is the least invasive, least expensive and least time-consuming treatment^5^. One of the disadvantages of current injection laryngoplasty techniques is their temporary enhancement, which frequently necessitates repeat injections over time.

In clinical settings, the use of calcium hydroxyapatite (CaHA), carboxymethylcellulose (CMC), hyaluronic acid, autologous fat and animal collagen are frequently described in the treatment of glottic insufficiency^6^. Except for CaHA, the majority of the materials have inconsistent resorption, and there is a chance of migration and granuloma formation^7–9^. Apart from the above-mentioned, specific injectable materials for vocal fold treatment remain scarce^10^. Although direct injection of acellular materials such as fat or gels can improve clinical outcomes, they only serve as a temporary filler at the paraglottic space. In contrast, it has been demonstrated that the incorporation of cells or biomolecules into acellular materials, such as hydrogel, provides better retention than acellular materials^11^. Therefore, tissue engineering strategies for designing an effective biomaterial for vocal fold treatment have been highlighted to facilitate local healing and restore their native function^12^. An example is the use of mesenchymal stem cells (MSCs) or secretome encapsulation in hydrogel to promote native tissue regeneration and reduce the local immune system^13^. With that, current study serves as a preliminary study to develop a hydrogel which has potential for cell encapsulation in future work, in providing regenerative properties.

Gelatin is derived from denatured collagen, a sustainable animal byproduct that possesses biocompatibility and biodegradable characteristics^14^. Due to their poor stability, crosslinkers are frequently necessary to improve their mechanical properties. Specifically, genipin and 1-ethyl-3-(3-dimethylaminpropyl)carbodiimide hydrochloride) (EDC) have been shown to be crosslinkers with low cytotoxicity and support stem cell differentiation to neuronal progenitor^15^. In addition, Wharton's jelly mesenchymal stem cells (WJMSCs) which is extracted from the umbilical cord, are favored in cell therapy due to their less invasive extraction method and lower infection risk^16^. These cells possess immunomodulatory and regenerative properties with great therapeutic potential^17^. Based on our literature review studies, there is lacking of in vitro evidence proving regenerative ability of WJMSCs in glottic insufficiency condition. Thus, the purpose of this study is to optimize the concentration of gelatin and genipin/EDC based on physicomechanical properties and develop a suitable hydrogel for injection into the vocal folds that can encapsulate WJMSCs as a potential treatment for patients with glottic insufficiency.

## Materials and methods

### Synthesis of genipin or EDC crosslinked gelatin hydrogels

Gelatin (250 Bloom; bovine bone origin) was supplied by Nitta Gelatin India Limited, Cochin, India. Genipin was purchased from FUJIFILM Wako Pure Chemical Inc., Osaka, Japan, while EDC and N-hydroxysuccinimide (NHS) were obtained from Sigma-Aldrich, St. Louis, MO, USA. Various gelatin concentrations ranging from 6% (w/v) to 10% (w/v) were utilized to create hydrogel. The gelatin was dissolved in DPBS at 50 °C. Upon obtaining a homogeneous solution, different concentrations of crosslinkers (genipin or EDC; both 0.1% (w/v)–0.5% (w/v)) were then added to achieve hydrogel crosslinking. The gelatin solution was mixed with genipin powder at 50 °C for three minutes to ensure homogenous polymerization until a yellow mixture was obtained. The fabrication method was demonstrated in Supplementary Vid. 1. EDC:NHS at a ratio of 4:1 was dissolved in ultrapure water and added to the gelatin solution. In calculations, all concentrations of hydrogel (%) represented as calculation of (w/v). Gelatin as "G" and genipin as "gn" were also used as abbreviations.

### Optimization of hydrogel formulation: gelation time, biodegradation, swelling ratio

The gelation time of 30 hydrogel groups (15 genipin-crosslinked gelatin and 15 EDC-crosslinked gelatin) were evaluated for their gelation time at 37 °C. The gelation of hydrogel was evaluated using the vial-tilting technique. The groups of hydrogels that achieved gelation within 20 min were evaluated for biodegradation rate and swelling ratio at 37 °C. The initial weight (W_i_) of hydrogel was determined and incubated in 0.0006% (w/v) collagenase Type I (Worthington, Lakewood, NJ, USA) for in vitro biodegradation test and PBS for swelling ratio test. The biodegradation rate was determined by measuring the final weight of hydrogel (W_f_) every 24 h, while the swelling ratio was measured at 30 min, 1, 2, 4, 6 and 8 h. The following was the formula for calculating the biodegradation rate and swelling ratio:

$$Bio\deg radation\;rate\left( {\frac{mg}{{hour}}} \right)\; = \;\frac{{W_{i} (mg) - W_{f} (mg)}}{Time\;(hour)}\; \times \;100\%$$

$$Swell{\text{in}} g\;ratio\left( \% \right)\; = \;\frac{{W_{f} (mg) - W_{i} (mg)}}{{W_{i} (mg)}}\; \times \;100\%$$

### Injectability and rheological property characterization: elasticity and viscosity

After fabrication process as mentioned in Section "Optimization of Hydrogel Formulation: Gelation Time, Biodegradation, Swelling Ratio", 1.0 ml of the liquid hydrogel was sucked in by a 1 ml syringe. It was allowed to polymerize at room temperature of 18 °C for 10 min. The injectability of the liquid hydrogel was demonstrated via 21G double bend needle (as used in current clinical setting) after polymerization. Rheological tests (TA Instrument Rheometer- AR2000) were used to further characterize the optimized hydrogel groups. At 37 °C, oscillation tests were conducted to determine the storage and loss moduli of the crosslinked hydrogel using parallel plates with a 20 mm diameter and a 1800 μm gap. All hydrogels were created between 20 and 22 h before the test. The commercial hyaluronic acid Juvederm®Ultra XC (Allergan, Pringy, France) was tested alongside the hydrogels for comparison. As described in Section "Synthesis of Genipin or EDC Crosslinked Gelatin Hydrogels", hydrogel in liquid form was freshly prepared for the flow test (viscosity). 500 μl of the solution was then applied to parallel plates separated by 500 μm gap. At 0.1% strain and an angular frequency range of 0.1–100 rad/s, both oscillation and flow test were conducted.

$${\text{Elastic}}\;{\text{modulus}}\; = \;{\text{Storage}}\;{\text{modulus }}{-}{\text{ loss}}\;{\text{modulus}}$$

### Crosslinking ratio

The hydrogels were lyophilized for 24 h using a freeze dryer (Ilshin, Korea). 10 mg of lyophilized hydrogel was weighed, and 200 μl of 10 × ninhydrin solution was added (Sigma-Aldrich, St. Louis, MO, USA). The solution was boiled for 2 min prior to the addition of 200 μl of 95% ethanol. The solution's absorbance was measured using a microplate reader (BioTek, PowerWave XS, Highland Park, IL, USA). The standard graph was plotted using serially diluted glycine solution as calibration purpose. The crosslinking ratio was calculated as follows:

$$crosslinking\;ratio\;(\% )\; = \;\frac{a - b}{a}\; \times \;100$$

where “a” denotes the concentration of free amino acid for non-crosslinked group, and “b” denotes the concentration of free amino acid for crosslinked groups.

### Contact angle

Hydrogels with flat surfaces were fabricated and 10 μl of distilled water was dropped onto the hydrogel. Using the ImageJ software (NIH, Bethesda, Maryland, United States), the contact angle was calculated after recording the droplet's angle.

### Water vapor transmission rate (WVTR)

WVTR was performed with minor modifications according to Salleh et al^18^. Prior to the test, the hydrogels were created in silicone moulds 20 to 22 h beforehand. The hydrogels were placed on top of a glass bottle containing 10 ml distilled water. The initial weight (W_i_) was measured before placing it in an environment with 5% CO^2^ and 37 °C. The final weight (W_f_) was recorded after 24 h, and the WVTR was calculated using the following formula.

$$WVTR\; = \;\frac{{W_{i} - W_{f} }}{A\; \times \;time}$$

where “A” is the surface area of glass bottle.

### Microporous structure

The hydrogel was fixed with 3% glutaraldehyde prior to serial ethanol dehydration. The lyophilized hydrogel was cut to obtain a cross-sectional view and coated with nanogold to analyze its pore size using field transmission scanning electron microscope (FESEM) (Supra 55VP, Zeiss, Obenkochen, Germany). ImageJ was used to calculate the pore size (NIH, Bethesda, MD, USA). The porosity was determined using the volume displacement method. We determined the initial weight (W_i_) and total volume (V) of lyophilized hydrogel. Then, the lyophilized hydrogel was submerged in absolute ethanol for 24 h at room temperature, and the final weight (W_f_) was determined. The porosity was computed using the formula:

$$Porosity\; = \;\frac{{W_{f} - W_{i} }}{\rho V}$$

where ρ = density of ethanol.

### Energy dispersive X-ray (EDX) analysis

Using EDX (Supra 55VP, Zeiss, Obenkochen, Germany), the element distribution on the hydrogel surface was analyzed. As a control, the non-crosslinked hydrogel was used.

### Fourier-transform infrared (FTIR)

At room temperature, a small sample of the lyophilized hydrogel was analyzed for its functional group at 4000 to 500 cm^−1^ with a resolution of 2 cm^−1^ per point at room temperature. This analysis utilized FTIR spectroscopy (Spectrum 400 FT-IR/NIR, PerkinElmer, Waltham, MA, USA). As a control, the non-crosslinked hydrogel was used.

### X-ray diffraction (XRD)

XRD was used to measure the crystallinity of the hydrogel. Bruker D8 Advance X-ray diffractometer equipment (D8 Advance, Bruker AXS GmbH, Karlsruhe, Germany) was used with a setting of 0.165 nm wavelength, 25 mA current and 40 kV voltage. A graph of 2-theta versus intensity (a.u.) was plotted.

### Isolation and culture of cells

Sample collection was done in a single tertiary center after obtaining institutional ethical approval from the Research Ethics Committee (UKM PPI/111/8/JEP-2020–818) based on Helsinki Declaration. All procedures in this study were conducted following guidelines and regulations of the ethical approval. Samples from the fetal segment of the umbilical cord (6–10 cm) were obtained from consenting healthy patients who were undergoing natural birth or elective surgery. All procedures in this study were performed in a laboratory with ISO 9001:2015 compliant facilities. The collected umbilical cord was disinfected with iodine and thoroughly washed. Arteries and veins were removed, and the umbilical matrix was fragmented. The sample was digested for 30 min to 1 h with 0.6% collagenase type I (Worthington, NJ, USA) at 37 °C incubator shaker. The sample was then washed with PBS and cultured with α-MEM (Gibco, CA, USA) supplemented with 10% fetal bovine serum (FBS) (Gibco, CA, USA). WJMSCs have been previously characterized by our colleagues in the same laboratory^19^.

### Cell seeding

Before analyzing cell-hydrogel interaction, cryopreserved WJMSCs were thawed and expanded for tests during passages 3 to 5. The cells were trypsinized at 70 to 80% confluence with 0.05% trypsin- EDTA (Gibco, CA, USA). In a biosafety cabinet, hydrogel production was performed under sterile conditions. On a hydrogel with a surface area of 452.4 mm^2^, a total of 50,000 cells in 20 ul culture medium were seeded.

### Cytotoxicity of hydrogel leachate

Leachate of hydrogel (2 g/ml) was prepared according to the ISO 10,993–12 protocol (Biological evaluation of medical devices-Part 12, fourth edition, 10.3 Extraction conditions and methods). WJMSCs were seeded at a density of 5000 cells/cm^2^ in a 48-well plate. After 24 h incubation in 5% CO^2^ and 37 °C, 200 ul of hydrogel leachate was added to triplicate wells, and the MTT assay was used to determine the cell activity after 48 h (Sigma-Aldrich, St. Louis, MO, USA). Briefly, leachate was extracted and cells were rinsed with PBS. In each well, 90 ul of pure medium with 10 ul of 5 mg/ml MTT solution were added, yielding a final concentration of 0.5 mg/ml MTT assay. After 4 h of incubation at 37 °C, 87.5 ul of the solution was removed and 100 ul of dimethyl sulfoxide (DMSO) was added (Sigma-Aldrich, St. Louis, MO, USA) to dissolve the formazan. Using a spectrophotometer (BioTek, PowerWave XS, Highland, IL, USA) to measure the absorbance of the final solution, the cell viability was calculated as follows:

$$Cell\;viability\; = \;\frac{{A_{t} }}{{A_{c} }}\; \times \;100\%$$

where A_t_ represents the absorbance of treatment groups and, A_c_ represents the absorbance of control group.

### Live and dead assay

On top of the hydrogel, a total of 50,000 WJMSCs were seeded, and cell viability was determined on day 2 and 7 using the LIVE/DEAD™ Viability/Cytotoxicity kit (Invitrogen, Waltham, MA, USA). First, the hydrogel was washed with sterile PBS, and then the PBS with the detached cells was collected to perform cell counting using trypan blue. Next, 200 μl of a mixture of calcein-AM and ethidium homodimer-1 mixture (ratio of 1:4) was added to the hydrogel and incubated for 30 min. The fluorescent cell was observed using Nikon Eclipse Ti fluorescence microscope (Nikon, Tokyo, Japan). The viability of the cell was calculated using ImageJ software (NIH, Bethesda, MD, USA). As described in Section "Microporous Structure", the morphology of these hydrogels seeded with cells was also analyzed using FESEM.

### Statistical analysis

All test data were analyzed using GraphPad Prism version 8.0 (GraphPad Software, Inc., San Diego, CA, United States). The differences between the control and treatment groups were statistically analyzed using one-way and two-way analysis of variance (ANOVA). The data was interpreted as mean ± standard deviation. In certain tests, 0.1% genipin was used as a control group because previous research demonstrated that this concentration was not cytotoxic^20^.

### Ethical approval

This study was approved by the Research Ethics Committee of Universiti Kebangsaan Malaysia (UKM PPI/111/8/JEP-2020–818). Informed consent was obtained from all subjects involved in this study.

## Results

### Genipin-crosslinked gelatin hydrogel exhibited favorable physical properties, elasticity and viscosity

**Figure 1:**
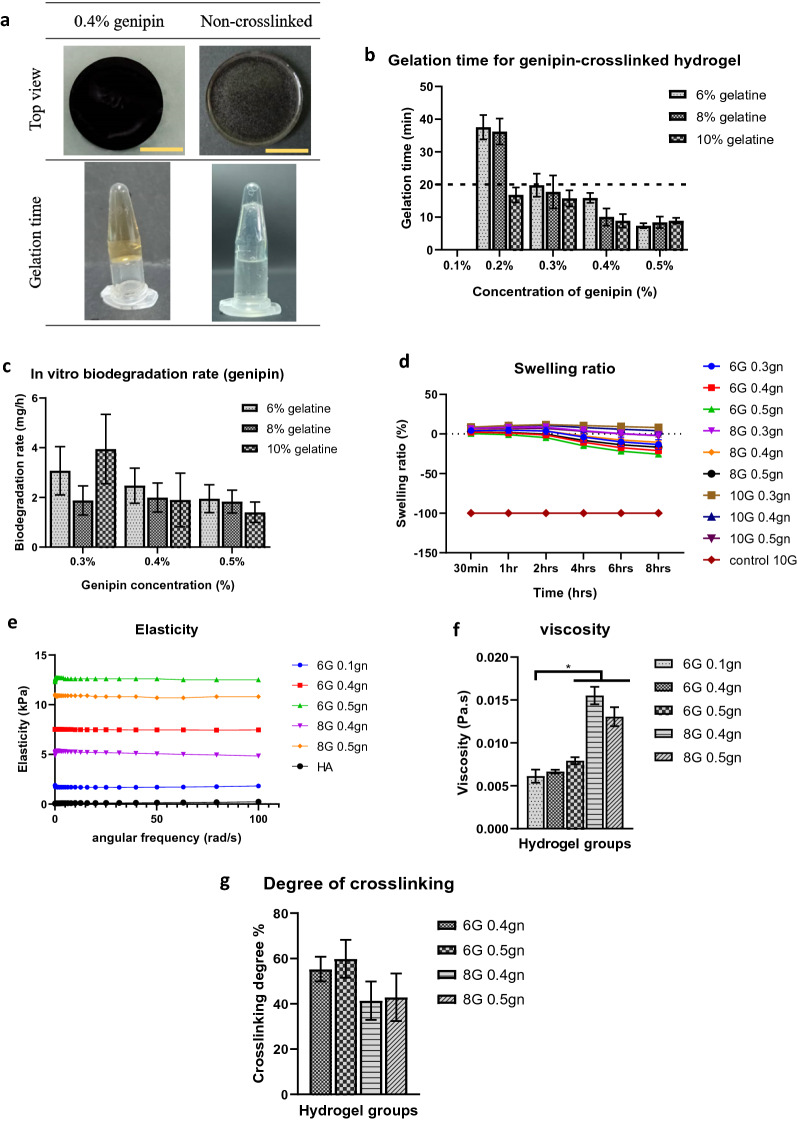
Optimization of the genipin crosslinked gelatin hydrogels (N = 3, n = 9, *p < 0.05). (**a**) Physical appearance of the 6% gelatin hydrogels. Each yellow scale bar represents 1 cm. (**b**) Gelation time of genipin-crosslinked gelatin hydrogels. (**c**) Biodegradation rate of genipin-crosslinked gelatin hydrogels. (**d**) Swelling ratio of genipin-crosslinked gelatin hydrogels. (**e**) Elasticity of the genipin-crosslinked gelatin hydrogels. Commercial hyaluronic acid was included as a comparison in this experiment. (**f**) Viscosity of the genipin-crosslinked gelatin hydrogels. (**g**) Crosslinking degree of genipin-crosslinked gelatin hydrogels.

In our study, gelatin hydrogel was initially crosslinked using genipin and EDC. After 24 h of crosslinking, genipin-crosslinked gelatin hydrogels appeared yellowish and turned blueish (Fig. 1a). In contrast, EDC-crosslinked gelatin hydrogels exhibited no color changes when compared to non-crosslinked gelatin hydrogels and appeared colorless (Supplementary Fig. 1a). The physical appearance of each crosslinked-gelatin hydrogel was consistent regardless of the gelatin and crosslinker concentrations. So, as a representative group, only the hydrogels with 6% gelatin and 0.4% crosslinkers were shown.

In our experiment, the hydrogel groups were selected based on an optimal gelation time of 20 min at 37 °C. This was used as a benchmark to select the hydrogel groups in our experiment as fast gelation could result in insufficient time for injection and slow gelation could cause hydrogel to leak into other tissue areas, or spaces after injection. All EDC-crosslinked gelatin hydrogel, except 0.1%, were able to achieve complete gelation within 20 min regardless of the gelatin concentration, whereas only gelatin hydrogels crosslinked with genipin concentrations of 0.3% and higher gelled completely within 20 min. Although crosslinking with EDC led to a faster gelation time than genipin (Fig. 1b, Supplementary Fig. 1b) in gelatin hydrogel, its in vitro degradation rate was significantly faster than that of genipin (Fig. 1c, Supplementary Fig. 1c). Our findings revealed that the EDC-crosslinked gelatin hydrogels degraded within 24 h, preventing their use over a longer study period (up to 7 days). Consequently, all EDC-crosslinked gelatin hydrogels groups were eliminated from our study, and all subsequent experiments were conducted solely with genipin-crosslinked gelatin hydrogels (6–10%G; 0.3–0.5%gn).

To prevent airway obstruction, the ideal injectable hydrogel for vocal fold injection should exhibit a relatively low swelling ratio. Our results showed that, across all genipin-crosslinked gelatin hydrogel groups, a lower swelling ratio was associated with a lower gelatin concentration and a higher genipin concentration (Fig. 1d). Therefore, hydrogel formulations of 6% and 8% gelatin crosslinked with 0.4% and 0.5% genipin were selected for further characterization.

The injectability of the genipin-crosslinked gelatin hydrogels via 21G needle were performed after 10 min of fabrication at room temperature of 18 °C (Supplementary Vid. 1). 6G 0.1gn, 6G 0.4gn and 6G 0.5gn were observed to have watery-form hydrogel while, 8G 0.4gn and 8G 0.5gn were jelly-form hydrogel. However, all of the hydrogel can be injected after 10 min of polymerization in room temperature of 18 °C. The rheological properties of the hydrogel should mimic the elasticity and viscosity of the target native tissue in the vocal fold. Herein, our results showed that a higher elastic modulus was associated with a higher genipin concentration, indicating that the gelatin hydrogel was more rigid than flexible (Fig. 1e). In fact, every hydrogel formulation chosen from previous experiments (6G 0.4gn, 6G 0.5gn, 8G 0.4gn, 8G 0.5gn) exhibited a higher elastic modulus than the control (6G 0.1gn) and commercial hyaluronic acid, Juvederm® Ultra XC (Allergan, Pringy, France). Elastic modulus decreased with increasing gelatin concentration, and this effect was consistent in hydrogels crosslinked with genipin concentration (6G 0.4gn vs. 8G 0.4gn; 6G 0.5gn vs. 8G 0.5gn; Fig. 1e). In contrast, the elastic modulus increased when a higher concentration of genipin was utilized (6G 0.4gn vs. 6G 0.5gn; 8G 0.4gn vs. 8G 0.5gn; Fig. 1e). Alternatively, the viscosity of the hydrogel increased with increasing gelatin concentration irrespective of genipin concentration (6G 0.4gn vs. 8G 0.4gn; 6G 0.5gn vs. 8G 0.5gn; Fig. 1f). Overall, our results showed that genipin-crosslinked gelatin hydrogels can be made more elastic by combining a lower gelatin concentration with a higher genipin concentration. Increasing the gelatin concentration, on the other hand, will make the hydrogel more viscous.

The crosslinking ratio was determined to comprehend the crosslinking efficacy of different genipin concentrations and to justify its relationship with other physical tests. All of the selected groups exhibited a similar crosslinking ratio, according to our findings. However, when a higher gelatin concentration was employed, the crosslinking ratio decreased marginally (6G 0.4gn vs. 8G 0.4gn; 6G 0.5gn vs. 8G 0.5gn; Fig. 1g).

### Microporous structure and hydrophilicity of genipin-crosslinked gelatin hydrogels

**Figure 2:**
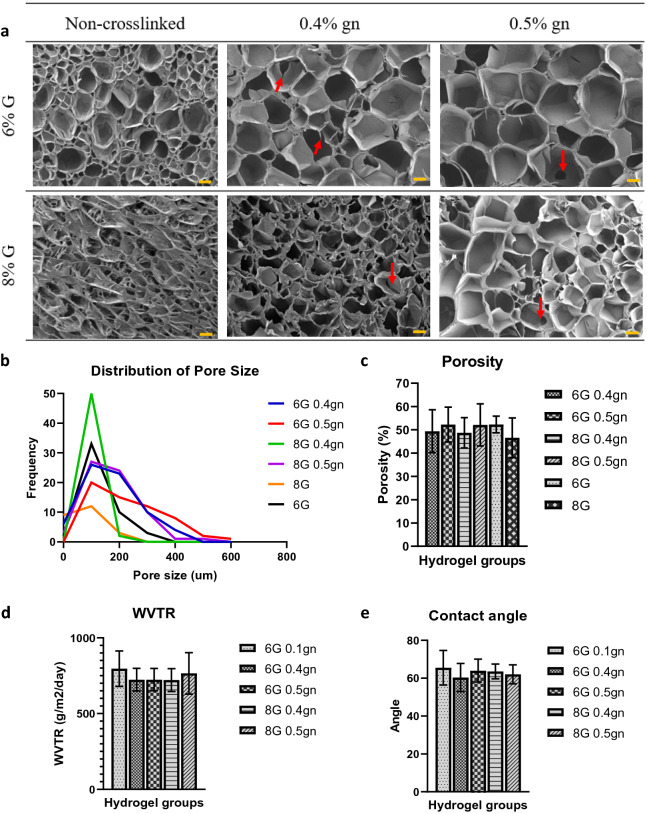
Microporous structure of the genipin-crosslinked gelatin hydrogels. (**a**) Scanning electron microscopy of the gelatin hydrogel (cross-sectional view) at 50 × magnification. Red arrow indicates interconnected pores. Each yellow scale bar represents 100 μm. (**b**) Pore size distribution of the genipin-crosslinked gelatin hydrogels. (**c**) Porosity of the genipin-crosslinked gelatin hydrogels. (**d**) WVTR of the genipin-crosslinked gelatin hydrogels. (**e**) Water contact angle of genipin-crosslinked gelatin hydrogels.

Optimal pore size and porosity are essential for nutrient absorption and angiogenesis. Previous research demonstrated that hydrogels with a porosity of 60% and pore size ranging from 120 to 325 μm were able to support cell growth and migration^21,22^. In our study, genipin crosslinking altered the morphology of the gelatin hydrogel pores, making them more spherical and larger (Fig. 2a). The 6G 0.4gn, 6G 0.5gn and 8G 0.5gn hydrogel groups had the largest pore size between 400 and 600 μm (Fig. 2b). In addition, all hydrogel groups exhibited a low porosity value (45% to 55%) (Fig. 2c). These values were consistent with the SEM image, which revealed fewer interconnected pores.

WVTR of more than 70 g/m^2^/day has been reported to support cell growth^23^. No matter the gelatin and genipin concentrations, the WVTR of all selected genipin-crosslinked gelatin hydrogels were consistent, as demonstrated by the results presented here (Fig. 2d). Besides, the WVTR results of these hydrogels were also comparable with the control (6G 0.1gn). In addition, the WVTR results of these hydrogels were comparable to those of the control group (6G 0.1gn). Next, the contact angle of a scaffold is a parameter used to determine a scaffold's hydrophilicity. Hydrophilicity of the scaffold is vital to enable cell interaction and adhesion. In this study, all hydrogels exhibited contact angles less than 90° and no statistically significant differences were observed among the genipin-crosslinked gelatin hydrogel groups (Fig. 2e). Collectively, these results supported the hydrophilicity of our hydrogel scaffold, indicating its capacity to promote cell adhesion.

### Genipin-crosslinked gelatin hydrogel possessed similar chemical characteristics with non-crosslinked gelatin hydrogel

**Figure 3:**
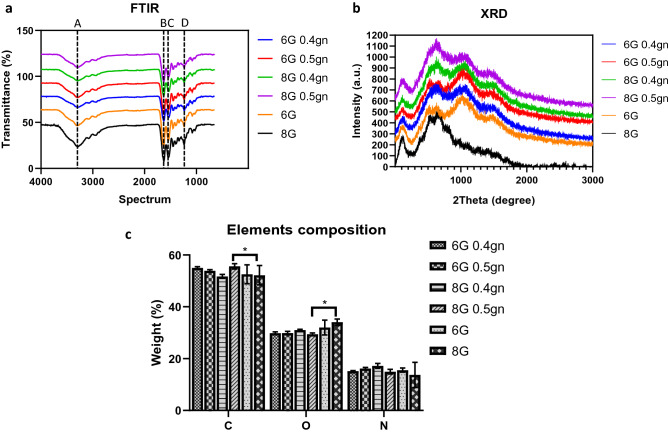
Chemical analysis of genipin-crosslinked gelatin hydrogels (*p < 0.05). (**a**) FTIR spectrum of the genipin-crosslinked and non-crosslinked gelatin hydrogels. (**b**) XRD spectrum of the genipin-crosslinked and non-crosslinked gelatin hydrogels. (**c**) Element distribution of carbon, oxygen and nitrogen in the genipin-crosslinked and non-crosslinked gelatin hydrogels.

Functional groups of gelatin should be retained, especially amide groups to serve as arginylglycylaspartic acid (RGD) receptor for cell adhesion and interaction. Four intensities were observed for the genipin-crosslinked and non-crosslinked hydrogel (Fig. 3a). Regions A and B were represented by hydroxyl groups, which serve as crosslinking sites for genipin^24^. The intensities of regions C and D also represented the nucleophilic attack initiated by genipin on the amide groups of gelatin^25^. The peak with the greatest intensity (consisting of the most non-crosslinked gelatin) was 8G, 6G, 6G 0.5gn, 8G 0.4gn, 8G 0.5gn, and 6G 0.4gn. Although the amide groups were reduced in genipin-crosslinked hydrogel, the functional groups were retained. All crosslinked hydrogels possessed an amorphous structure (Fig. 3b and Table 1), and their crystallinities were comparable to that of their non-crosslinked counterparts (6% G and 8% G). The amorphous structure of gelatin should be remained as this properties favours cell interaction. EDX was examined to understand the changes of element composition in gelatin after genipin crosslinking. Most chemical elements on the hydrogel were comparable between genipin-crosslinked and non-crosslinked gelatin hydrogels, with the exception of a specific group (8G 0.5gn) that exhibited a significant decreased of carbon element and significant increased of oxygen when compared to the non-crosslinked gelatin hydrogel (Fig. 3c).

**Table 1:** Crystallinity and amorphous percentage of the non-crosslinked and genipin-crosslinked gelatin hydrogels.

Set	Crystallinity (%)	Amorphous (%)
6G	18.0	82.0
8G	24.4	75.6
6G 0.4gn	14.8	85.2
6G 0.5gn	13.8	86.2
8G 0.4gn	13.6	86.4
8G 0.5gn	14.8	85.2

### Genipin-crosslinked gelatin hydrogels supported WJMSCs growth

**Figure 4:**
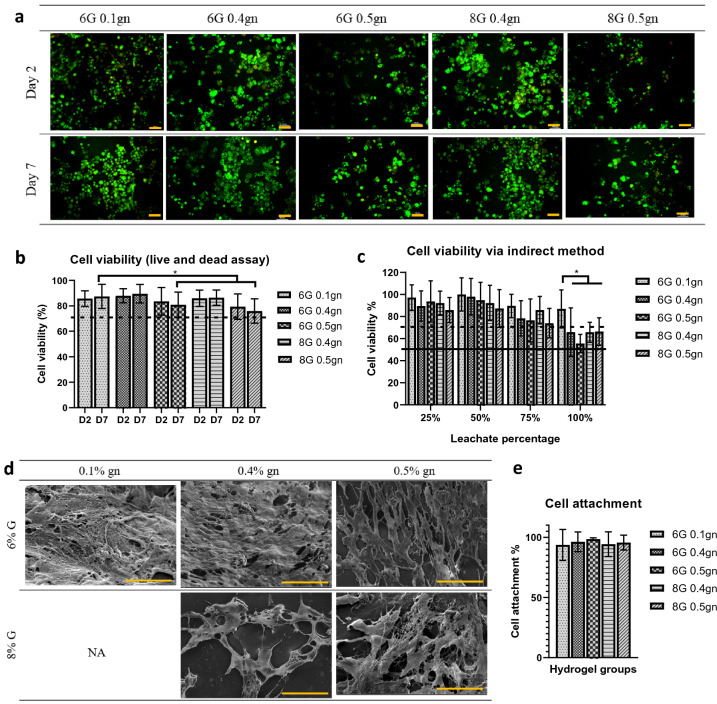
WJMSCs interaction with genipin-crosslinked gelatin hydrogels (N = 3, n = 9, *p < 0.05). (**a**) WJMSCs image of live and dead assay which were seeded on top genipin-crosslinked gelatin hydrogels at Day 2 and Day 7. (**b**) Cell viability of WJMSCs which were seeded on top genipin-crosslinked gelatin hydrogels at Day 2 and Day 7. (**c**) Indirect effect of genipin-crosslinked gelatin hydrogels towards the viability of WJMSCs as determined by MTT assay. (**d**) SEM morphology of the hydrogel scaffolds seeded with WJMSCs. (**e**) Cell attachment of the WJMSCs on the genipin-crosslinked gelatin hydrogels at Day 2. Each yellow scale bar represents 100 μm.

For injectable hydrogels to be translated into future clinical applications, their safety must be a primary consideration. To determine the effect of our hydrogels on the viability of WJMSCs, WJMSCs were seeded directly onto the hydrogel scaffolds. All seeded cells displayed a spherical morphology, and their viability exceeded 70%, as determined by our findings (Fig. 4a, b). On day 7 of WJMSCs culture, the cell viability of 6G 0.5gn and 8G 0.5gn were significantly lower than that of the 6G 0.1gn control (Fig. 4b), which could be attributed to the higher concentration of genipin used. In addition, we adapted an indirect method utilizing leachate medium to determine the effect of our hydrogels on the viability of WJMSCs. Our results demonstrated that all concentrations of hydrogel leachates, with the exception of 100% pure hydrogel leachates, exhibited a viability of 70% or greater in WJMSCs (Fig. 4c). 100% leaching solution administration resulted in lower cell viability than our control (6G 0.1gn).

In terms of cell attachment, the structure of WJMSCs on 6% gelatin hydrogel exhibited a spindle-like morphology. In contrast, WJMSCs seeded on 8% gelatin hydrogel exhibited a stellate-like morphology (Fig. 4d). Intriguingly, this distinct morphology was observed in both 6% and 8% gelatin hydrogels, irrespective of their genipin concentration. Despite this, each hydrogel group exhibited greater than 90% cell attachment (Fig. 4e). These findings hence corroborated with our previous findings that a hydrophilic hydrogel was optimal for cell adhesion.

## Discussion

The widespread application of tissue engineering includes the regeneration of skin wounds, cartilage, bone, smooth muscle and nerves. However, evidence regarding the development of injectable hydrogel for vocal fold regeneration is still lacking. Current treatment focuses only on promoting vocal fold augmentation, and not tissue regeneration. Therefore, this study serves as a preliminary investigation on development of an injectable genipin-crosslinked gelatin hydrogel formulation that can potentially be used to deliver cells or biomaterials for the regeneration of vocal folds.

Naturally derived gelatin has been shown to be biocompatible and biodegradable. However, gelatin has a significant disadvantage in that its mechanical stability is inferior and it liquefies at 37 °C (physiological temperature)^26^. Chemical crosslinkers such as genipin and EDC has been described to increase the mechanical strength of gelatin. Genipin is a crosslinker with non-zero length, whereas EDC is a crosslinker with zero length^27^. Despite the fact that EDC has a shorter gelation time than genipin, our results demonstrated that genipin-crosslinked gelatin hydrogel was more resistant to rapid degradation than EDC, which was consistent with the findings of a previous study^15^. This finding may be explained by the differential crosslinking mechanism of genipin and EDC, wherein genipin forms intermolecular links (slower but stronger bonds) and EDC forms intramolecular links (faster but weaker bonds)^24^. Due to its high biodegradation rate and low mechanical strength at 37 °C (physiological temperature), we concluded that EDC-crosslinked gelatin hydrogel was unsuitable for injection into the vocal folds, which were subsequently excluded from the study. Furthermore, our genipin-crosslinked gelatin hydrogel exhibited a low swelling ratio, which was ideal for injections into the vocal folds to prevent edema from exudate absorption^28^.

The storage modulus was greater than the loss modulus for all of the genipin-crosslinked gelatin hydrogels in this study, indicating that they were all elastic. The elastic modulus of these genipin-crosslinked gelatin hydrogels was greater than that of commercial hyaluronic acid (Juvederm® Ultra XC). Juvederm® Ultra XC had been commercially used for soft tissue augmentation, but its rapid biodegradation could be attributed to its rheological characteristics^29^. The degree of crosslinking of gelatin significantly influenced its storage modulus^30^. Overall, the elasticity and storage modulus of genipin-crosslinked gelatin hydrogels can be increased by combining a lower gelatin concentration with a higher genipin concentration. In contrast, a higher gelatin concentration can result in a higher hydrogel viscosity and a lower crosslinking ratio. This phenomenon may be explained by the fact that the bulky chemical structure of gelatin at higher concentrations results in a shielding effect of lysine residue during the genipin crosslinking reaction, thereby reducing the crosslinking efficiency and resulting in a lower crosslinking ratio^31^. Intriguingly, the stiffness of the hydrogel was found to influence the expression of chondrogenic, osteogenic and angiogenic properties^32^. The storage modulus of both 0.4% genipin-crosslinked gelatin hydrogels (6G 0.4gn and 8G 0.4gn) fell within the range of 2 to 10 kPa, had potential to facilitate the differentiation of MSCs into a neural or myocyte lineage. Furthermore, the higher viscosity observed in hydrogels with higher gelatin concentrations can be attributed to the stronger physical interaction between gelatin chains, which made gelation easier^33^. Considering that 6% gelatin-based hydrogels (6G 0.4gn and 6G 0.5gn) had a lower viscosity than 8% gelatin (8G 0.4gn and 8G 0.5gn), we anticipate that 6% gelatin-based hydrogels will be easier to inject into the vocal folds (as shown in Supplementary Vid 2).

The incorporation of genipin into the gelatin hydrogel did not alter the chemical characteristics of gelatin. Gelatin is an organic substance rich in amino acids that is primarily composed of carbon, oxygen and nitrogen^34^. Gelatin's primary functional groups are composed of carboxylic, amide, ester, aromatic and alkane^35^. Data from previous study suggested that a high crystallinity biomaterial demonstrated a high degree of stiffness, which may in turn inhibit cell attachment^36^. Nonetheless, our results demonstrated a lower intensity of amide peak upon genipin induced gelatin crosslinking via the free amide group, but the amorphous structure remained, which may facilitate the growth of MSCs.

All 0.4% genipin-crosslinked hydrogels (6G 0.4gn and 8G 0.4gn) demonstrated excellent cell viability (70%) with WJMSCs, indicating the safety of these hydrogel formulations for cell encapsulation as a tissue-engineered product for potential vocal fold treatment. On day 7, the cell viability of WJMSCs cultured on 0.5% genipin-crosslinked hydrogel (6G 0.5gn and 8G 0.5gn) was lower than that of the control (6G 0.1gn). This could be attributed to the higher concentration of genipin used to crosslink the hydrogels, which increased cytotoxicity. As the genipin-crosslinked gelatin hydrogels exhibited hydrophilic properties, the cells were able to adhere to the hydrogel's surface, as demonstrated by the attachment of WJMSCs to our hydrogels. However, the morphology of WJMSCs varied depending on the gelatin hydrogel concentration. WJMSCs on 6% gelatin hydrogels exhibited a spindle-like morphology, indicating a tendency for maturation, whereas WJMSCs on 8% gelatin hydrogels exhibited a stellate-like morphology, possibly due to the tendency for osteocyte differentiation^37^. On the other hand, senescent MSCs appeared larger and to have more pseudopodia, whereas young MSCs resembled fibroblasts^38^. In light of these results, additional characterization research should be conducted to confirm our hypothesis regarding the state of WJMSCs and their morphology on our genipin-crosslinked gelatin hydrogels. In addition, the ability of WJMSCs to maintain viability on the manufactured hydrogel may also be explained by the ability of the crosslinked hydrogels to transmit nutrients, as they had an acceptable WVTR value and pore size ranging from 100 to 400 μm. It has been shown that the pore size of hydrogel influences cell signaling^39^. A higher porosity scaffold is associated with improved cell adhesion and differentiation^40^, even though there is currently no conclusive evidence on the optimal porosity for cell signaling. Indeed, Suresh et al. reported that hydrogels with a 60% porosity were capable of promoting mesenchymal stem cells derived from bone marrow^21^. In comparison, the porosity of our genipin-crosslinked hydrogels had a lower interconnected porous network, ranging from 45 to 55% porosity, while the viability of WJMSCs remained above 70%. Based on our current results, we urge that further testing like migration, proliferation and the inflammatory cytokine profiling are necessary to elucidate the cellular response during WJMSCs encapsulation in the genipin-crosslinked gelatin hydrogel.

## Conclusion and future outlooks

We concluded that 6G 0.4gn and 8G 0.4gn were two of the best genipin-crosslinked gelatin hydrogel formulations because they achieved complete gelation within 20 min, exhibited a relatively low swelling ratio, degraded more slowly in vitro, had an elastic modulus between 2 and 10 kPa and supported WJMSCs viability and attachment after 7 days of in vitro culture. It should be noted, however, that these formulations were recommended based on the in vitro physical properties and cell viability experiments that we had conducted thus far. Additional experiments, such as proliferation, migration and inflammatory response, must be conducted to prove its suitability for vocal fold augmentation. Despite this, the work presented here serves as a preliminary study that permits the exploration of additional strategies, such as WJMSCs encapsulation and drug delivery, in addition to our optimized formulations. These strategies will pave the way for the development of a promising treatment for vocal fold regeneration.

## Supplementary Information

Supplementary Information 1.

## Data Availability

The dataset generated and/or analyzed during the current study are available from the corresponding author on reasonable request.
