# Supplementary material for: In vitro evaluation of genipin-crosslinked gelatin hydrogels for vocal fold injection
Source: Sci Rep. 2023 Mar 29;13:5128. doi: 10.1038/s41598-023-32080-y (PMC10060255; doi:10.1038/s41598-023-32080-y)
Supplement: Supplementary file 1 — Supplementary Information 1. [file 41598_2023_32080_MOESM1_ESM.pdf]

Supplementary Fig. 1

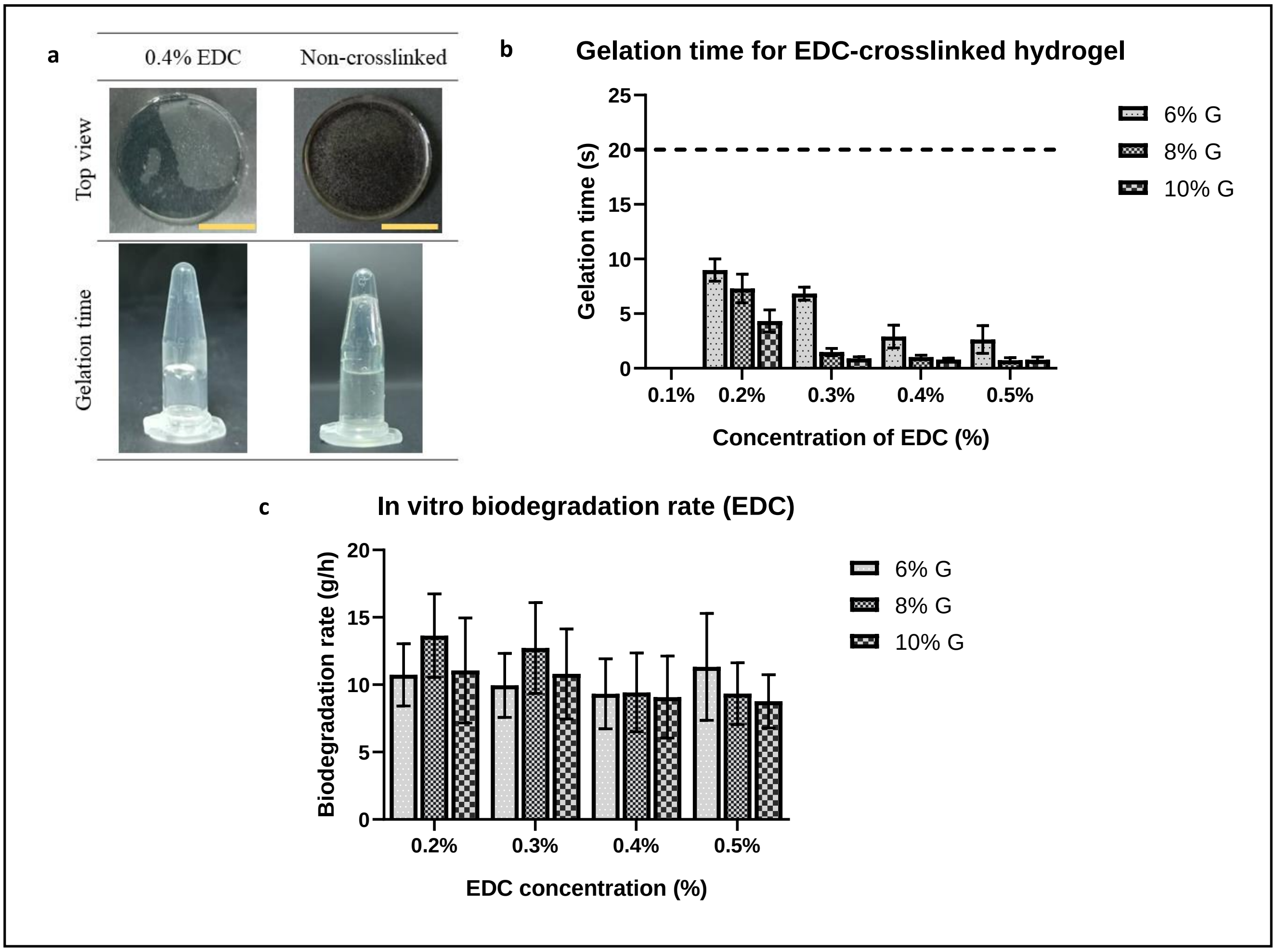

Supplementary Fig 1: Optimization of the EDC crosslinked gelatin hydrogels (N=3, n=9, *p<0.05).
(a) Physical appearance of the 6% gelatin hydrogels. Each yellow scale bar represents 1 cm.
(b) Gelation time of EDC-crosslinked gelatin hydrogels.
(c) Biodegradation rate of EDC-crosslinked gelatin hydrogels.

Supplementary Vid. 1

https://drive.google.com/file/d/1EnmauS7YsdAeV91F0FE0cw536XAqvPjE/view?usp=sharing

Supplementary Vid. 1: Fabrication of genipin-crosslinked gelatin hydrogel.

Supplementary Vid. 2

https://drive.google.com/file/d/1mveuJdPl06uhcJ-zkvwgYMVtTss34-ny/view?usp=sharing

Supplementary Vid. 2: Injectability of genipin-crosslinked gelatin hydrogel.
